# Biological nitrification inhibition by root exudates of native species, *Hibiscus splendens* and *Solanum echinatum*

**DOI:** 10.7717/peerj.4960

**Published:** 2018-06-19

**Authors:** Chelsea K. Janke, Laura A. Wendling, Ryosuke Fujinuma

**Affiliations:** 1 School of Agriculture and Food Sciences, University of Queensland, Australia; 2 Current affiliation: Department of Natural Sciences, International Christian University, Tokyo, Japan; 3 Current affiliation: VTT Technical Research Centre of Finland Ltd, Espoo, Finland

**Keywords:** Native species, Nutrient cycling, Nitrogen, Biological nitrification inhibition, Ecology, Root exudates

## Abstract

Australian native species grow competitively in nutrient limited environments, particularly in nitrogen (N) limited soils; however, the mechanism that enables this is poorly understood. Biological nitrification inhibition (BNI), which is the release of root exudates into the plant rhizosphere to inhibit the nitrification process, is a hypothesized adaptive mechanism for maximizing N uptake. To date, few studies have investigated the temporal pattern and components of root exudates by Australian native plant species for BNI. This study examined root exudates from two Australian native species, *Hibiscus splendens* and *Solanum echinatum,* and contrasted with exudates of *Sorghum bicolor*, a plant widely demonstrated to exhibit BNI capacity. Root exudates were collected from plants at two, four, and six weeks after transplanting to solution culture. Root exudates contained three types of organic acids (OAs), oxalic, citric and succinic acids, regardless of the species. However, the two Australian natives species released larger amount of OAs in earlier development stages than *S. bicolor*. The total quantity of these OAs released per unit root dry mass was also seven-ten times greater for Australian native plant species compared to *S. bicolor*. The root exudates significantly inhibited nitrification activity over six weeks’ growth in a potential nitrification assay, with *S. echinatum* (*ca*. 81% inhibition) > *S. bicolor* (*ca*. 80% inhibition) > *H. splendens* (*ca*. 78% inhibition). The narrow range of BNI capacity in the study plants limited the determination of a relationship between OAs and BNI; however, a lack of correlation between individual OAs and inhibition of nitrification suggests OAs may not directly contribute to BNI. These results indicate that Australian native species generate a strongly N conserving environment within the rhizosphere up to six weeks after germination, establishing a competitive advantage in severely N limited environments.

## Introduction

Nitrogen (N) and phosphorous (P) are generally the most commonly limiting nutrients in terrestrial ecosystems, depending on ecosystem development and input processes (Agren, Wetterstedt & Billberger, 2012; Menge, Hedin & Pacala, 2012). In particular, the mobility of N in soils can limit the recovery of this nutrient by plants in both natural and anthropized landscapes. A key adaptation strategy for plants in N-limited soils is biological nitrification inhibition (BNI) mediated by root exudates from plants to increase the residence time of N in soil. Biological nitrification inhibition has been identified in a range of plant species, including grasses, weeds, and agricultural crops (Subbarao et al., 2007a; Sun et al., 2016; O’Sullivan et al., 2016; O’Sullivan et al., 2017). Plants exhibiting BNI function are often species originated and/or adapted to N poor soils, suggesting that BNI in plants is an adaptive mechanism for growth in N-limited environments (Lata et al., 2004; Subbarao et al., 2007a). Subsequently, BNI has been suggested as a competitive growth strategy that gives advantage to several invasive species such as *Andropogon gayanus* and *Bromus tectorum* (Subbarao et al., 2007a; Rossiter-Rachor et al., 2009; Blank & Morgan, 2012). Weed species including, *Lolium rigidum*, *Bromus driandrus*, *Raphinus raphinastrum*, and *Avena fatua*, have also demonstrated BNI, which is associated with their vigorous growth (O’Sullivan et al., 2017).

With the exception of some native African grasses (Subbarao et al., 2007a), to date there has been very little investigation of BNI activity in plant species native to N-poor soils. Indigenous plant species are a key element for rehabilitation of degraded soils and in ecosystem restoration initiatives. Native plants that demonstrate BNI may provide a robust option for ecosystem rehabilitation and restoration where soils are N-limited, requiring minimal maintenance inputs. Many native Australian plants are adapted for growth in areas of highly variable rainfall, and in a wide variety of nutrient-impoverished soils requiring a range of strategies to suit growth environments (McKenzie et al., 2004). Given their adaptation to growth in N-deficient soils and contrasting climates, Australian native plants may actively release compounds that inhibit nitrification via root exudates.

The plant-derived compounds involved in BNI from root exudates are not well identified. Several compounds in *Sorghum bicolor* root exudates including, 3-(4 hydroxyphenyl) propionate (MHPP), sakuranetin, and sorgoleone have been identified as active compounds for BNI (Subbarao et al., 2013; Zakir et al., 2008). Additionally, 1,9-decanediol in rice root exudates has been noted as having inhibitory effects on nitrification (Sun et al., 2016). The difficulty in identifying active compounds likely originates from the involvement of multiple compounds to form BNI function. Subbarao et al. (2007b) proposed that a minimum of three types of compounds exuded from plant roots are involved with BNI, and that these are likely anionic in nature. Organic acids (OAs), in their anionic form, have been widely implicated in the regulation of nutrients within plant rhizospheres (Kollmeier et al., 2001; Ryan & Delhaize, 2001; Walker et al., 2003) through interaction with soil microbes and the solid phase (Jones et al., 2003), however their role in BNI has not been previously examined. Organic acids may contribute to a portion of the BNI function of root exudates in plants, partially fulfilling the proposed requirement of three or more compounds that confer inhibitory properties in BNI (Subbarao et al., 2007b).

The objective of this study was to examine the root exudates from selected Australian native plant species for BNI activity, and to analyse these root exudates to identify and quantify anionic OAs potentially involved in BNI. This study did not test for previously identified BNI-active compounds, as the primary aim was to conduct a preliminary screening for the role of OAs in BNI, to the exclusion of other compounds of which some are likely present in root exudates. A glasshouse nutrient growth medium system was designed to test the hypotheses: (1) root exudates of selected Australian native plant species inhibit the rate of nitrification; and (2) the BNI activity of root exudates is correlated with total concentrations, or specific types, of anionic organic acids.

## Materials and Methods

### Selection of plant species

Two native Australian plant species, *Hibiscus splendens* and *Solanum echinatum*, were selected because of their adaptation to slightly acidic, light textured soils with low soil N content, as determined by the CSIRO soil map (National Land & Water Resources Audit, 2001). These soil properties have been previously correlated with plants which exhibited BNI capacity (Subbarao et al., 2012; Zhu et al., 2012), leading to the supposition that Australian native plant species adapted to similar environments may also release root exudates that inhibit soil nitrification processes. *H. splendens* is known as a hardy Australian native shrub and is not considered to be at risk. It is commonly found throughout the east coast of Australia growing along forest margins and in disturbed or cleared areas. *S. echinatum* is an annual or short-lived perennial herb native to northern Australia. Its typical habitat includes rocky outcrops or nearby sandy and alluvial deposits. Like *H. splendens*, *S. echinatum* is not considered to be at risk (classified as a plant of least concern in Northern Territory, Australia). *S. bicolor* was examined as a positive control due to documented BNI by its root exudates (Subbarao et al., 2007a).

### Growth in solution culture

Four germinated seedlings of each species were transplanted to a solution culture system. The solution nutrient content was adjusted to respective nutrient concentrations similar to those typically found in soils (Asher & Blamey, 1987), with a modification of NH_4_-N concentration at six percent and a solution pH of 5.6 (Table 1). The nutrient solutions were replaced with freshly prepared nutrient solution every week, and phosphorus (P) was added every second day to prevent P deficiency. Three replicates (four plants per replicate) of the solution culture system per plant species were arranged using a completely randomised design in glasshouse conditions with temperature maintained at a constant 26–28 °C, and light levels according to ambient night-day sunlight regime of June–July in Southeast Queensland, Australia (*ca*. 10.5 h day^−1^).

**Table 1:** Chemical composition of nutrient solution culture.

**Element**	**Salts**	**Final element concentration (µM)**
Ca	CaCl_2_.2H_2_O	1,073
	CaSO_4_.2H_2_O	
N	Ca(NO_3_)_2_.4H_2_O	742
	NH_4_NO_3_	45
K	K_2_SO_4_	300
Mg	MgSO_4_.7H_2_O	95
S	Provided in Mg, Ca, K, Zn, Mn and Cu Salts	344
Fe	Na_2_FeEDTA	6
B	H_3_BO_3_	1
Zn	ZnSO_4_.7H_2_O	0.5
Mn	MnSO_4_.H_2_O	0.5
Cu	CuSO_4_.5H_2_O	0.2
Mo	Na_2_MoO_4_	0.01
P	KH_2_PO_4_	5
P	KH_2_PO_4_	K=1 *P* = 1

### Root exudate collection and laboratory analyses

Plant root exudates were collected following two, four, and six weeks of growth using methods similar to those previously utilized in root exudate studies (Subbarao et al., 2006; Zakir et al., 2008; Zhu et al., 2012). Four intact plants (an entire treatment unit of replicate) were removed from nutrient solution and rinsed with deionized water before placing the plant roots in an aerated collection bottle containing 500 mL of 1 mM NH_4_Cl (Zakir et al., 2008). Plant root exudates were accumulated for 24 h then the plants were returned to fresh nutrient solution. Each of the 500 mL samples were concentrated to 10 mL by passing the root exudate solution through 20 g of prepared anion exchange resin (Bio-Rad AG1 Anion Exchange resin, 100–200 mesh; Bio-Rad, Hercules, CA, USA) based on protocols in Kollmeier et al. (2001) and Ma et al. (2002). The 10 mL concentrates were then evaporated to dryness at 45 °C under a fume hood followed by resuspension in 20 mL of methanol. A 5 mL aliquot of each methanol-exudate solution was reserved for OA identification using ultra-high performance liquid chromatography-ultraviolet (UHPLC-UV) analysis. The remaining 15 mL of each sample was evaporated and resuspended in 200 µl dimethyl sulfoxide (DMSO). Dimethyl sulfoxide was used in order to recover any water insoluble compounds potentially exuded by plant roots. The samples were stored at 4 °C prior to further testing.

### Identification of exudates

Organic acids in sub-samples of each exudate replicate were determined using UHPLC-UV and UHPLC-mass spectroscopy (MS) analyses at the Queensland Department of Science, Information Technology and Innovation (Dutton Park, QLD). The two UHPLC methods utilized were based on parameters detailed in Dionex (2004), Ibáñez & Bauer (2014) and Phenomenex (2015). Analysis by HPLC-UV identified these compounds following protonation; the compounds exuded from plant roots, collected, and used in the soil assay incubation were in the deprotonated, anionic form. Thus, all references to oxalic, citric and succinic acid were assumed to be present as oxalate, citrate, and succinate, respectively, in soil solution.

### Nitrification inhibition tests

Nitrification inhibition by collected root exudates was examined using a nitrification potential assay at pH 7.2 (Hart et al., 1994) in a red, clay Ferrosol, pH_w_ 5.8 and nitrifying potential *ca*. 0.63 mg NO_3^−^_ L^−1^ h^−1^, collected from the Darling Downs (South-east Queensland, Australia). Six grams of field-moist soil with 40 mL of NH_4^+^_ substrate solution, comprised of 3.8 mM (NH_4_)_2_SO_4_ made with 1 mM phosphate buffer solution at pH 7.2, was placed into a 100 mL flask. Concentrated exudate samples in 200 µL DMSO were re-suspended in 1 mL deionised (DI) water and added to the soil-slurry. A negative control was included, containing 200 µL DMSO made up to 1 mL with DI water to eliminate the effects of DMSO from the evaluation. Flasks were sealed with Parafilm® with three pin holes per flask to allow gas exchange but prevent moisture loss. All flasks were placed on an orbital shaker incubator at 180 rpm and 25 °C. Samples of soil slurry were collected at times between 0 and 24 h, then centrifuged (3000 rpm for 10 min) to separate solid and liquid phases. The supernatant was filtered to 0.45 µm and stored at −20 °C until further analysis. The concentrations of NO_2^−^_ in soil slurry supernatants were determined colorimetrically using sulphanilamide and N-(1-naphthyl)-ethylene-diamine dihydrochloride (10-107-05-1-A, Lachat Instruments, 2008); the concentrations of NO_2^−^_ + NO_3^−^_ in soil slurry supernatants were measured using a Cd reduction method (10-107-04-1-A, Lachat Instruments, 2008). Then the concentration of NO_3^−^_ was estimated as the difference between the concentrations of NO_2^−^_ + NO_3^−^_ and NO_2^−^_.

### Data processing and statistical analyses

Nitrification potential (mg N L^−1^) was calculated based on the difference between initial (0 h) and subsequent (22 h) measured concentrations of NO_3_-N. Negative values for nitrification potential were removed from the analysis and figures. The end time of incubation was determined by the concentration of NO_3_-N in control as the method assumes a linear increase of NO_3^−^_. In this study, the linear increase of NO_3_-N was observed until 22 h (data not shown); hence, 22 h data were treated as the end-point samples. Nitrification measurement at time zero allowed accurate interpretation of NO_3_-N production by removing potential discrepancies associated with varying concentrations of indigenous NO_3_-N in the test soil. The nitrification rates were calculated as the average concentration (mg N L^−1^), minus the initial concentration (0 h), divided by the number of hours in incubation. The extent of BNI was estimated as the percent reduction in nitrification rate of soil incubated with exudates, compared to nitrification rates in untreated soil (control). Organic acid production by individual plants (mg plant^−1^ day^−1^) was determined using concentration data (mg L^−1^) multiplied with the concentrated sample volume (0.02 L), and then divided by the number of plants per pot. Two-way analysis of variance (ANOVA) was used to determine the effects of plant species and age on NO_3_-N values in soil incubations, both with root exudates and without (control included). Post-hoc Tukey pairwise comparison was used to determine grouping of significant effects at *p* = 0.05. To determine influencing factors without the masking effect of control values, both production of NO_3_-N and BNI capacity from soil incubated with root exudates (control excluded) were analysed by two-way ANOVA with post-hoc Tukey analysis for the effects of plant species and age.

## Results

### Effect of root exudates on nitrification

**Figure 1:**
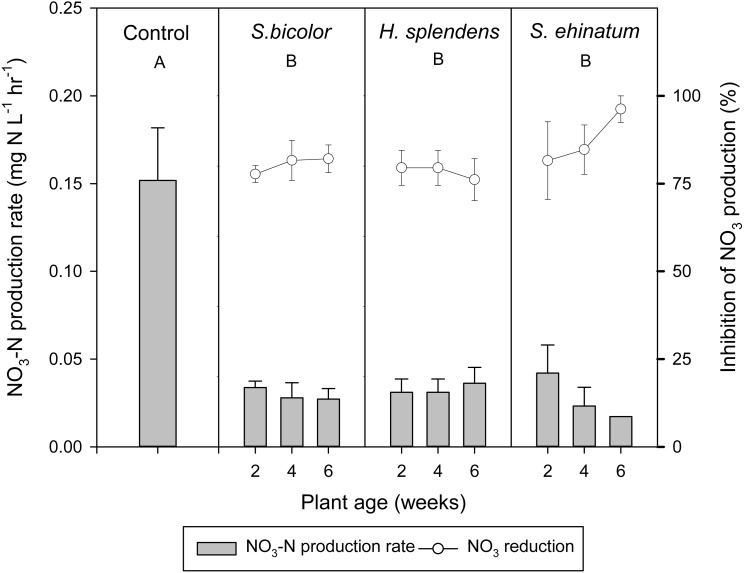
Nitrate-N (NO_3_-N) concentration in Ferrosol extracts. Nitrate-N concentration in extracts of Ferrosol incubated with selected plant root exudates for 22 h. Error bars indicate a standard error of mean. Capital letters show significant differences (*p* = 0.05) between production of NO_3_-N in untreated soil and soil treated with exudates of each plant species.

The exudates of both *S. bicolor* and the Australian native plant species significantly inhibited nitrification rates (*p* < 0.05) in soil solution, relative to the uninhibited soil (Fig. 1). Over six weeks of growth, nitrification rates were limited to a range of 0.0145–0.0440 mg NO_3_-N L^−1^ h^−1^ in the presence of *S. bicolor* exudates; 0.0183–0.0539 mg NO_3_-N L^−1^ h^−1^ with *H. splendens* exudates; and 0.0047–0.0581 mg NO_3_-N L^−1^ h^−1^with *S. echinatum* exudates. Excluding control values, no significant variation in effect on overall nitrification rates was identified between the tested plant species, with *H. splendens* demonstrating slightly higher nitrification rates (*ca*. 0.0328 mg NO_3_-N L^−1^ h^−1^), than *S. echinatum* (*ca*. 0.0285 mg NO_3_-N L^−1^ h^−1^). The positive control, *S. bicolor*, exhibited a nitrification rate intermediate between the two Australian native species (0.0296 mg NO_3_-N L^−1^ h^−1^) (Fig. 1).

The BNI activity, indicated as the percent reduction in nitrification rates in soil treated with root exudates, varied with plant species and age but not significantly (Fig. 1). Over six weeks, exudates from *S. bicolor* demonstrated intermediate BNI activity (80%), with *S. echinatum* exudates exhibiting slightly stronger inhibition (81%) than that of *H. splendens* (78%) (Fig. 1). Whilst variation of BNI activity in response to plant age occurred, this was not statistically significant (*p* > 0.05). All three plant species demonstrated effective BNI at age two, four, and six weeks, with exudates of *S. echinatum* exhibiting the greatest effect on soil nitrification over six weeks.

### Organic acids

**Figure 2:**
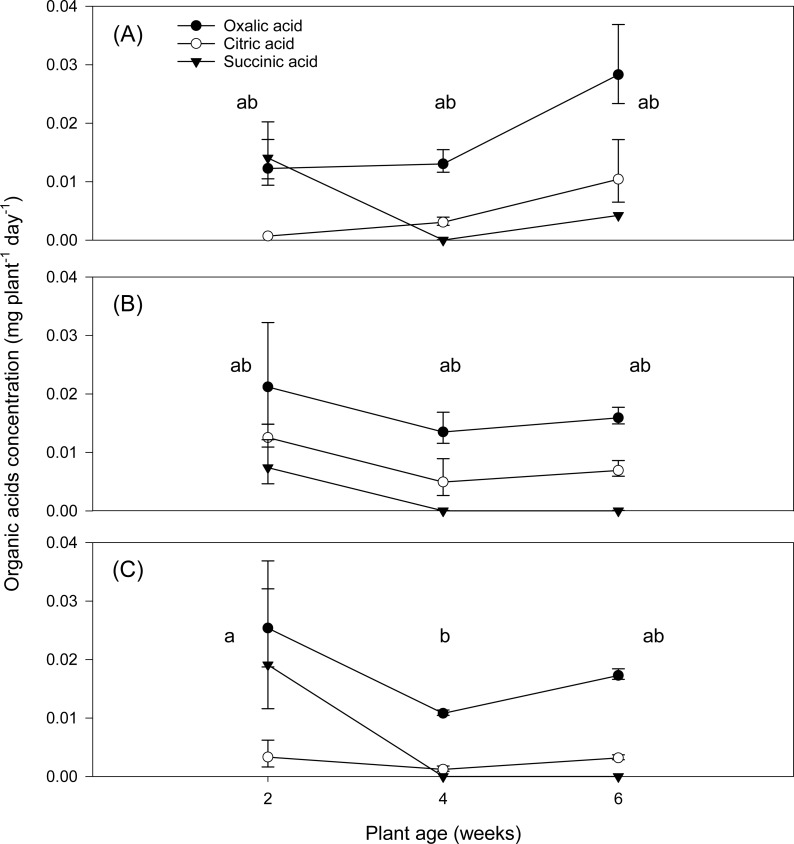
Organic acid exudation by plant species over time. Mean concentration of organic acids (mg plant^−1^ day^−1^) identified in root exudates from (A) *Sorghum bicolor*, (B) *Hibiscus splendens* and (C) *Solanum echinatum* as a function of time. Lowercase letters indicate significant variation in total OA release as a function of the plant species and age interaction (*p* < 0.05). Each error bar indicates a standard error of mean.

Three aliphatic OAs, oxalic, citric and succinic, were identified in the root exudates of species examined herein. *S. bicolor* exuded the greatest quantity of the OAs examined, yielding a total of 3.7% more OA than *S. echinatum* and 26.2% more than *H. splendens* (Table 2). On a dry root mass basis, however, *S. echinatum* released the greatest total quantity of OAs, producing *ca.* 943% more OAs than *S. bicolor* and 40% more than *H. splendens*, over the six-week growth period. Whilst the release of total OAs from *S. bicolor* (Fig. 2A) and *H. splendens* (Fig. 2B) did not significantly vary over six weeks, OA concentration in root exudates of *S. echinatum* (Fig. 2C) did decrease significantly from two to four weeks, but recovered to intermediate levels by six weeks.

**Table 2:** Organic acid concentration and quantity. Mean concentration and the mean quantity per dry root mass of detected organic acids (OAs) over the 6 week growth period, from the root exudates of selected plant species.

Species	Oxalic acid	Citric acid	Succinic acid^a^	Total
Concentration (µg L^−1^)				
*Sorghum bicolor*	36	9	37	82
*Hibiscus splendens*	34	16	15	65
*Solanum echinatum*	36	5	38	79
OA/dry root mass (mg g^−1^)				
*Sorghum bicolor*	0.05	0.01	0.02	0.07
*Hibiscus splendens*	0.32	0.16	0.05	0.52
*Solanum echinatum*	0.49	0.07	0.17	0.73

**Notes.**

a Succinic acids were detected in first 2 weeks only.

**Figure 3:**
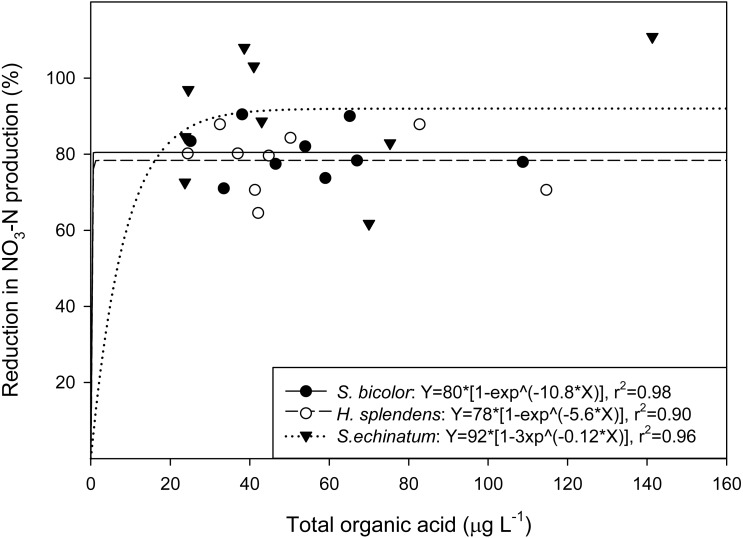
Correlation of total organic acid concentration to nitrification inhibition. Total organic acid concentration in root exudates of each plant species tested as a function of net BNI capacity.

**Figure 4:**
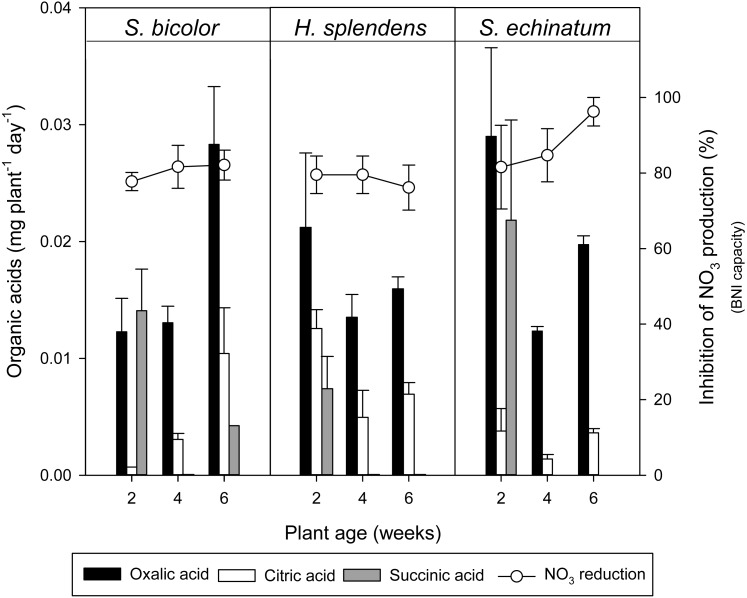
Individual organic acids and nitrification inhibition. Relationship of individual organic acid concentrations in root exudates of each plant species to net NO_3_-N reduction. Each error bar indicates a standard error of mean.

An exponential relationship was identified between total OA concentration and BNI capacity (Fig. 3), with approximately 98%, 90%, and 96% of the reduction in NO_3_-N production correlated to the OA content of root exudates of *S. bicolor*, *H. splendens*, and *S. echinatum*, respectively. However, individual organic acids in root exudates from each of the plant species did not show a strong relationship to their corresponding BNI capacity (Fig. 4).

## Discussion

### Biological nitrification inhibition by root exudates

Root exudates of the Australian native species *H. splendens* and *S. echinatum* demonstrated substantial BNI as indicated by significantly reduced rates of nitrification in the soil assay (Fig. 1). Previous research has demonstrated the capacity for BNI by several plant species (Subbarao et al., 2007a; Sun et al., 2016; O’Sullivan et al., 2016; O’Sullivan et al., 2017); however, the present study provides the first evidence for substantial BNI capacity by root exudates of Australian native plant species. As a class of plants that grow competitively in Australia’s nutrient-poor soils, control of N supply through BNI may provide a competitive advantage for establishment by maintaining N in NH_4^+^_ form for longer periods.

Although statistically insignificant, a difference in the inhibitory effects between the two Australian plant species (Fig. 1) was observed, with higher nitrification rates detected in soil treated with root exudates of *H. splendens* over six weeks’ growth (Fig. 1) compared to *S. echinatum*. Substantial variation in the magnitude of BNI has been demonstrated in root exudates from different species of pasture grasses and legumes (Subbarao et al., 2007a) and rice (Tanaka, Nardi & Wissuwa, 2010; Sun et al., 2016), suggesting that the development of BNI capability within plant species and varieties may vary a great deal. Functionally and/or geographically grouped plant species (i.e., those with similar adaptive environments) may exhibit variation in BNI activity from root exudates.

Plant age did not significantly affected the BNI activity of root exudates from the plant species examined, however substantial variation was seen between and within the study plants over a six week period (Figs. 1 and 4). It is hypothesized that the effect of plant age may become more apparent over a longer period of growth, as has been identified in previous studies (Zakir et al., 2008; Subbarao et al., 2013).

The findings reported herein support the hypothesis that the selected Australian native species, adapted to growth in a diverse range of low-N environments, release root exudates that inhibit soil nitrifying microorganisms.

### Exudation of organic acids

Of the three organic acids identified in plant root exudates, oxalic acid release was relatively similar in all three plants, with variation in the citric and succinic acids (Fig. 2; Table 2). Oxalic and citric acids in root exudates have both been associated with nutrient availability (Strom, 1997; Pan et al., 2016), particularly the availability of P (Fox & Comerford, 1990; Wouterlood, Lambers & Veneklass, 2005), through effects on soil pH and associated biological processes. Succinic acid exudation has been less widely reported, having a largely unknown function, but has been identified under P-stressed conditions (Gaume et al., 2001) and in forest soils (Fox & Comerford, 1990). Australian soils are generally severely P-limited due to the geological age of the continent; hence, the greater release of succinic acid at early growth phases may be an additional adaptive trait for P stress.

Exudation of total OAs from roots of the Australian native species examined demonstrated a general decline with time (Fig. 2) in comparison to *S. bicolor*. The relatively greater OA exudation at an early growth stage by the Australian native species may be an adaptive trait whereby young plants access maximum N resources through root exudation for competitive establishment in N-limited environments. This is further supported by biometric data (Figs. S1 and S2, Table S1) which indicated, after two weeks’ growth, that both Australian native species had substantially less above-ground and root system biomass compared with *S. bicolor*. Root exudation represents a metabolic cost to plants that may have otherwise utilized carbon in excreted compounds for growth (Walker et al., 2003).

Where the total release of OAs over six weeks from *S. bicolor* (82 µg L^−1^) and *S. echinatum* (79 µg L^−1^) were similar, *H. splendens* released much less at 65 µg L^−1^ (Fig. 2; Table 2). However, the total OAs were comprised of different relative proportions of the three identified OAs (Fig. 4). Furthermore, on a dry root mass basis, *S. echinatum* and *H. splendens* released more than seven times the quantity of OA per gram dry root compared to *S. bicolor* (Table 2). This comparatively greater release of OAs from Australian native species suggests that the quantity of total OAs exuded by plant roots is not dependant on root biomass, but rather is dictated by some other mechanism. Root exudation is known to modulate in response to various factors such as plant species, root system architecture, age of plant, pH, soil texture and compaction, water stress, and nutrient stress or toxicity (Kollmeier et al., 2001; Ryan & Delhaize, 2001; Walker et al., 2003).

### Relationship of organic acids to biological nitrification inhibition

Regression analysis between total OAs and BNI capacity (Fig. 3) indicates the quantity of OAs in root exudates is strongly correlated with inhibition of NH_4_-N oxidation. However, this high degree of correlation may be an artefact of the linear grouping of total organic acid values from *ca*. 20–80 µg L^−1^ over a limited BNI range (*ca*. 70–90%), which conversely suggests little correlation of OAs to BNI capacity (i.e., the reduction in NO_3_-N production was relatively similar despite increases in total OA). Investigation of root exudate OA content across a suite of plants exhibiting a range of BNI capacities is required to conclusively determine the relationship of OA to BNI. Analysis of the interrelationship between individual OA concentrations and NH_4_-N oxidation inhibition (Fig. 4) by each of the tested plant species, including plant age, did not show a clear relationship between a specific OA and BNI capacity. This suggests that although concentrations of total OAs correspond to increased inhibitory activity, individual OAs are not directly responsible for BNI.

Although untested in the present study, a proposed mechanism for inhibitory effects of total OAs is the chelation of soil solution copper (Cu). Copper is required for the activation of the ammonia-oxidising enzymatic pathway (Miller & Nicholas, 1985), and well-known inhibitors (allylthiourea and thiourea) are known to chelate metals (Bedard & Knowles, 1989). Exudation of OAs is a documented response to the presence of Cu in soil solution (Ahonen-Jonnarth et al., 2000) and has been linked to the tolerance of Cu (Murphy et al., 1999). However, this study did not test for Cu chelation and cannot therefore distinguish whether Cu chelation by anionic OAs or co-release of OAs with unidentified BNI compounds is responsible for the correlation of total OAs to BNI capacity (Fig. 3). In addition, whilst the present study did not screen for compounds other than anionic OAs in root exudates, it is likely that these compounds are also present in the tested exudates.

The release of OAs is likely a general response of establishing plants to enhance nutrient availability (Kollmeier et al., 2001; Ryan & Delhaize, 2001; Walker et al., 2003), which may contribute to BNI and/or coincide with the exudation of other compounds that are involved in BNI. The results presented do not support or negate the second study hypothesis; the limited variation in BNI in the tested plant species did not permit effective analysis of OA correlation to a range of BNI capacities. However, individual identified OAs were not strongly correlated with measured nitrification inhibition (Fig. 4), indicating that the effect of OAs on BNI, if any, may be indirect.

### Significance of BNI in native species

Understanding the role of BNI in establishment and persistence of native species is important when considering natural landscapes, ecological succession, and revegetation programs. Nitrogen availability is a major driver of succession in vegetation states (Hacker, Toole & Melville, 2011), with implications for landscape conservation and threatened plant species that are not able to regulate N availability. Extinction of native plant species, which do not exhibit a competitive N regulating strategy such as BNI, may irreversibly alter indigenous ecosystems, as other dependant organisms are not able to survive, or dominating plants (native or invasive) harbour predatory organisms (Dangremond, Pardini & Knight, 2010). Manipulation of soil N status significantly influences vegetation dynamics and may also contribute to the success of restoration programs (Hacker, Toole & Melville, 2011). Selection of native species for utilization of BNI function would require careful consideration and testing, as variation in BNI capacity between varieties of plant species has been noted. It is therefore important to understand the plant-soil interactions of root exudates from native species and their role in N cycling.

## Conclusions

This study has provided the first evidence for a high degree of BNI activity in root exudates of Australian native plant species *H. splendens* and *S. echinatum*. Organic acids, while present, may not have a direct and/or individual role in BNI function in root exudates of Australian natives. The previously unexamined class of plants provides a new category of plant species for investigation of the major active components and function of BNI. The present study identifies an active physiological process in native plants, which is an important consideration for native ecology.

##  Supplemental Information

10.7717/peerj.4960/supp-1 Supplemental Information 1 Analytical results of soil-inhibitor incubation assay over time Recorded measurements of NO_2_-N and NO_x_ to determine the level of nitrification activity. Measurements were taken over 0–24 h.

10.7717/peerj.4960/supp-2 Supplemental Information 2 Analytical results of exudate analysis for organic acids High liquid performance chromatography, using ultra-violet was used to determine the presence and concentration of organic acids in a sub-sample of root exudates. Raw results are presented in a table.

10.7717/peerj.4960/supp-3 Table S1 Biomass data Biomass data after 42 days of growth in nutrient solution culture. Table shows the mean wet and dry mass, and the mean moisture content, of each plant species.

10.7717/peerj.4960/supp-4 Figure S1 Plant growth monitoring Growth of the major stem of plant over 42 days. Measurements taken from the main stem at the highest node to the beginning of the root system.

10.7717/peerj.4960/supp-5 Figure S2 Plant root growth Growth of the root system over 42 days. Measurements taken from the tip of the longest root to the initiation of the root system from the stem. Data here is not indicative of total growth, refer to Table S1 for biomass data.

## Additional Information and Declarations

### Data Availability

The following information was supplied regarding data availability: The raw data are provided in the Supplemental Files.
