# Supplementary material for: Biological nitrification inhibition by root exudates of native species, Hibiscus splendens and Solanum echinatum
Source: PeerJ. 2018 Jun 19;6:e4960. doi: 10.7717/peerj.4960 (PMC6014310; doi:10.7717/peerj.4960)
Supplement: Supplemental Information 2 — High liquid performance chromatography, using ultra-violet was used to determine the presence and concentration of organic acids in a sub-sample of root exudates. Raw results are presented in a table. [file peerj-06-4960-s002.docx]

**Organic acids analysis, Job-15-0668**

Method details:

Instrument: UHPLC Thermo fisher Ultimate 3000

Detection: UV @ 210nm

Column: HPLC Acclaim Organic Acid (OA) 150x2.1mm 3um

Mobile phase: 20mM KH2PO4 adjusted to pH 2.7 with H3PO4

Flow rate: 0.15mL/min.

Column temp: 45

Post column temp: 25

Injection volume: 1uL

Table 2: Sample results in mg/L.

| sample | Oxalic | Citric | Succinic |
| --- | --- | --- | --- |
| MeOH | n.d. | n.d. | n.d. |
| 15-0668-0001 | 2.08 | 0.14 | 2.432 |
| 15-0668-0002 | 1.706 | n.d. | 4.198 |
| 15-0668-0003 | 3.579 | n.d. | 1.816 |
| 15-0668-0004 | 1.817 | n.d. | 0.623 |
| 15-0668-0005 | 6.123 | 2.833 | 2.511 |
| 15-0668-0006 | 4.784 | 2.185 | 1.307 |
| 15-0668-0007 | 2.421 | 0.534 | 4.044 |
| 15-0668-0008 | 6.26 | 0.155 | 1.113 |
| 15-0668-0009 | 6.537 | 1.297 | 6.298 |
| 15-0668-0010 | 2.645 | 0.703 | n.d. |
| 15-0668-0011 | 2.102 | 0.41 | n.d. |
| 15-0668-0012 | 3.08 | 0.729 | n.d. |
| 15-0668-0013 | 1.925 | 1.775 | n.d. |
| 15-0668-0014 | 3.111 | 1.02 | n.d. |
| 15-0668-0015 | 3.072 | 0.178 | n.d. |
| 15-0668-0016 | 2.178 | 0.276 | n.d. |
| 15-0668-0017 | 2.264 | 0.115 | n.d. |
| 15-0668-0018 | 2.03 | 0.338 | n.d. |
| 15-0668-0019 | 7.307 | 3.566 | n.d. |
| 15-0668-0020 | 5.796 | 0.904 | n.d. |
| 15-0668-0021 | 3.887 | 1.782 | 0.848 |
| 15-0668-0022 | 3.209 | 1.003 | n.d. |
| 15-0668-0023 | 3.534 | 1.492 | n.d. |
| 15-0668-0024 | 2.817 | 1.662 | n.d. |
| 15-0668-0025 | 3.189 | 0.673 | n.d. |
| 15-0668-0026 | 3.579 | 0.72 | n.d. |
| 15-0668-0027 | 3.59 | 0.513 | n.d. |

n.d. = not detected.

Analysis date: 03/09/2015

Provider: DSITI, Science delivery, Chemistry Centre.
