# Supplementary material for: Biological nitrification inhibition by root exudates of native species, Hibiscus splendens and Solanum echinatum
Source: PeerJ. 2018 Jun 19;6:e4960. doi: 10.7717/peerj.4960 (PMC6014310; doi:10.7717/peerj.4960)
Supplement: Table S1 — Biomass data after 42 days of growth in nutrient solution culture. Table shows the mean wet and dry mass, and the mean moisture content, of each plant species. [file peerj-06-4960-s003.docx]

**Supplemental Table S1** - Biomass data after 42 days of growth in nutrient solution culture. Table shows the mean wet and dry mass, and the mean moisture content, of each plant species

|  | **Wet Mass (g)** | **Dry Mass (g)** | **Moisture Content (g)** | **Moisture Content (%)** |
| --- | --- | --- | --- | --- |
|  | **Upper plant biomass (stem & leaves)** | | | |
| ***S. bicolor*** | 37.37 | 5.63 | 31.75 | 84.94 |
| ***H. splendens*** | 11.25 | 1.31 | 9.94 | 88.33 |
| ***S. echinatum*** | 6.99 | 1.09 | 5.90 | 84.41 |
|  | **Lower plant biomass (roots)** | | | |
| ***S. bicolor*** | 54.07 | 4.75 | 49.32 | 91.21 |
| ***H. splendens*** | 11.16 | 0.63 | 10.53 | 94.32 |
| ***S. echinatum*** | 14.57 | 0.44 | 14.13 | 96.96 |
