# Supplementary figures and images for: Biological nitrification inhibition by root exudates of native species, Hibiscus splendens and Solanum echinatum
Source: PeerJ. 2018 Jun 19;6:e4960. doi: 10.7717/peerj.4960 (PMC6014310; doi:10.7717/peerj.4960)

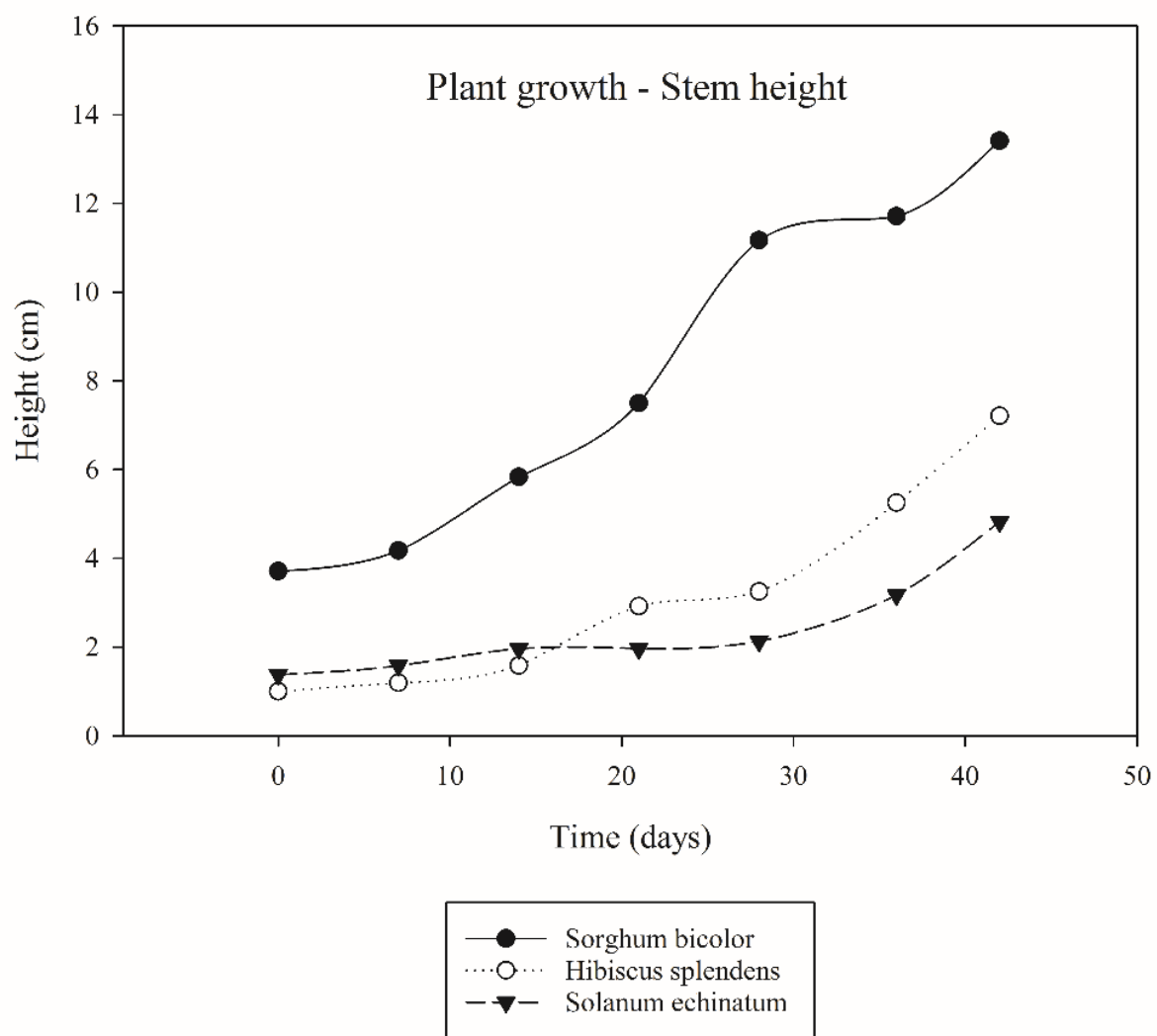

Supplement: Figure S1 — Growth of the major stem of plant over 42 days. Measurements taken from the main stem at the highest node to the beginning of the root system. [file peerj-06-4960-s004.pdf]

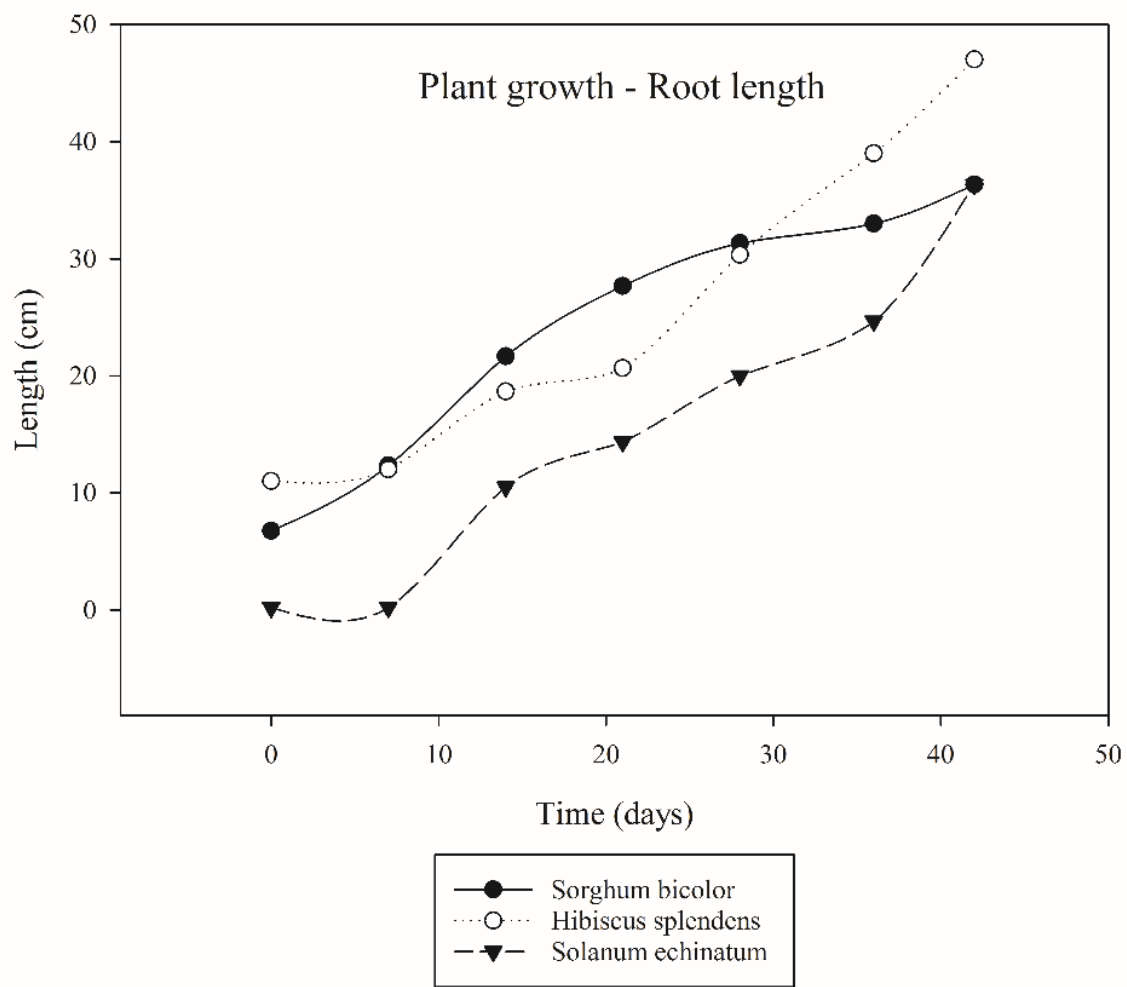

Supplement: Figure S2 — Growth of the root system over 42 days. Measurements taken from the tip of the longest root to the initiation of the root system from the stem. Data here is not indicative of total growth, refer to Table S1 for biomass data. [file peerj-06-4960-s005.pdf]
